# Handbook of the Diseases of the Eye and Their Treatment

**Published:** 1885-01

**Authors:** 


**Handbook of the Diseases of the Eye and their Treatment.** By HENRY R. SWANZY, A. M., M. B. F. R. C. S.; Ophthalmic Surgeon to the Adelaide Hospital, Dublin. With illustrations. New York: D. Appleton & Co., 1, 3 and 5 Bond street. 1884.

This handbook is intended for students in ophthalmology, and is among the best in this specialty that we have perused. The author is a elear writer and practical ophthalmologist, and gives here very practical instruction in this important department of medical science.

